# Digital Twin and Smart Manufacturing in Industries: A Bibliometric Analysis with a Focus on Industry 4.0

**DOI:** 10.3390/s22041388

**Published:** 2022-02-11

**Authors:** Georgiana Moiceanu, Gigel Paraschiv

**Affiliations:** 1 Department of Entrepreneurship and Management, University POLITEHNICA of Bucharest, 060042 Bucharest, Romania; georgiana.moiceanu@upb.ro; 2 Department of Biotechnical Systems, University POLITEHNICA of Bucharest, 060042 Bucharest, Romania

**Keywords:** digital twin, smart manufacturing, Industry 4.0, bibliometric analysis

## Abstract

Technology is being used in our society in all areas, mostly in industry, and generates the most interest in current research since it is a part of day-to-day activities. The main objective of this research was to use bibliometric analysis to analyze the production of scientific literature on digital twin and smart manufacturing with a focus on Industry 4.0, using information from the Web of Science database. To conduct the study, the keywords necessary for data selection were chosen, and then analyzed based on different variables such as author productivity, citations, most productive institutions, publishers with the highest number of publications, scientific document classification, countries with the highest number of publications, and a network analysis using VOSviewer. The results showed Tao F. and Soderberg R. were the main authors, that China was the country with the highest knowledge, and Elsevier was the main publisher. Although the subject has only been in publication for five years, digital twin will constitute an important part of future technologies due to its rapid ascension, proof of this being its yearly productivity (2020 producing the highest number of materials). Papers published in 2021 were excluded, but the difference between the numbers of materials found and those analyzed shows that 2021 will be even more productive than 2020.

## 1. Introduction

Technology expansion and consolidation are key components of all activities, whether concerning natural science or industry. New challenges facing competitive markets impose a new approach, thus digitalization is seen as an opportunity for industries, because using digitalization in manufacturing can achieve higher productivity [1]. Taking advantage of the new technologies in constant development will ensure companies from different industries maintain their competitive edge in an increasingly competitive market.

Since the advent of the first computers, the first element of technology, people have tried to abstractly model the different problems they were facing, with the intention of finding answers using processing power. In recent times, industries have focused on the new concept of digital twin [2]. According to research, digital twin offers professional products and services and a partnership responsible for the total digitization of design and manufacturing processes [3,4,5,6]. Digital twins are currently being developed for every process, product, or service in a company, in order to simulate all kinds of scenarios before their commencement in the physical world.

Due to exponential technological advance, highlighted by storage capacity and processing power, applications have contributed to the development of the fourth technology revolution known as Industry 4.0 (a set of technological principles aimed at taking full advantage of the new technologies). This allowed for an easy and rapid connection between people, assembly lines, machines, robots, and processes, etc., [7].

According to [8], the number of publications on digital twin show a rapid growth from around 2016, then a doubling of growth every year.

Smart manufacturing could optimize manufacturing procedure and the entire business process in terms of achieving higher productivity, while lowering costs and waste material [9]. The once-known process of manufacturing, making goods to be sold and bought, human labor, tools, equipment, etc., is now a smart process due to the digital transformation of manufacturing. The implication of “smart” technologies is aligned with the principles of the fourth industrial revolution [10]. Additionally, integration of the manufacturing system both on a vertical and horizontal level is smart manufacturing from the facility level [11]. To classify the smart manufacturing system, scientists use sustainability and asset utilization [12]. Since the use of smart manufacturing can be applied in various industries, a series of elements such as technologies, factors and characteristics need to be known. Even though smart manufacturing is a huge leap forward for companies, specially tailored methodologies are constantly being developed for many industries, allowing even greater optimization to take place.

When connecting the terms digital twin and smart manufacturing, it is observed that digital twin is an important part of smart manufacturing innovation [13]. The merger of digital twin and smart manufacturing services could make important changes in the usage, product design, and other types of processes [9,14,15,16,17]. Data streams are constantly being analyzed through digital twin, thus, the results may present trends in the actual performance of the manufacturing process. The comparative analysis that can be performed can help optimize the manufacturing process in the real world [18]. Virtual models of physical objects created using digital twin can help simulate their behavior, thus understanding of their state, and estimating and analyzing the dynamic changes [17,19,20]. In this way, constant optimizations in the design phase, in a virtual environment, eventually lead to the direct production of the target product, without discovering mistakes later in the production phase that otherwise would cost enormous quantities of money.

By adding the term Industry 4.0 to the database search of publications and scientific data, it can be observed that the appearance of Industry 4.0 and technical resource possibilities have enabled the progress of digital twin, and thus smart manufacturing [21].

This is a normal process, because of a single factor: disruption coming from new companies. Industry 4.0 can help start-ups leverage the advantage of traditional original equipment manufacturer (OEMs), by directly implementing the concepts of digitalization, cloud, smart factory, and smart manufacturing [22,23,24,25,26,27]. Industry 4.0 closed the gap between these large companies and newcomers, so that every company in every industry moves towards these concepts, to maintain their competitive edge, but more importantly, secure their future in their respective market.

The end users are receiving the best from both worlds: they receive optimized products to best fit the market’s needs, while procuring them at a cheaper price due to competition, and companies achieve cost savings by producing error-free products.

The construction industry is heavily affected by challenges due to low productivity and poor technology adoption. The authors of [28] analyzed the current state of DT concept and adoption in the construction industry, showing that there is a high potential for the use of digital twins to solve numerous challenges in the construction industry.

The use of digital twins is heavily increasing within smart cities, due to the rapid development of connectivity and internet of things (IoT). A growing number of cities are becoming “smart”, thus communities benefit from the use of smart city digital twins. Another benefit is that the use of digital twins will determine research for advanced AI algorithms [29].

The importance of digital twins in the construction industry was also described by [30], who noted that while digital twins enable the bi-directional flow of data between digital and physical entities and consequently change each other, digital shadows represent a physical model with only one-way data flow. Despite particular challenges, digital twin technologies are gaining popularity in various sectors, and the greatest pull for this technology is coming from industry [31,32].

Smart buildings require expensive operation and maintenance, outlining the need for digital twins, created from building intelligent management systems to improve these operations [33]. This can lead to beneficial interactions between users, the digital twin and the application framework, with the purpose of lowering maintenance costs.

Digital twins and smart manufacturing have also begun to make an impact in smart prognostics and health management systems [34]. Interconnectivity and the large gathering of data helps to detect faults. The use of smart prognostics and health management systems applies to any manufacturing operation across industry. Data acquisition, preparation and analysis, and modeling lead to better predictions and deployment.

The issue of health diagnosis and the benefits of digital twin have also been researched by [35], outlining the need for an intelligent health diagnosis and maintenance method for smart manufacturing, enabling maintenance personnel to identify significant influencing factors without knowing the health degradation of the equipment.

The authors researched the use of digital twin on the prognosis and health management of a proton exchange membrane fuel cell, reaching a high prediction accuracy, despite limited measurement data [36].

Even though smart manufacturing is being successfully implemented in different industries, research regarding the new 5G connectivity and its benefits are limited, according to [37]. In their paper they outlined the importance of Industry 4.0 and digital twin in reaching the potential of 5G networks.

Another research conducted a survey for the shipbuilding industry, investigating the adoption level of smart manufacturing and digital twins in eight shipyards from South Korea, proposing a new framework for smart shipyard maturity level assessment. Their results showed that adopting smart technologies for smart manufacturing led to better results than conventional methods [38].

Also the way DT and Industry 4.0 is changing the shipbuilding industry was discussed by researchers. Many issues that this industry faces generate losses, such as quality requirements, market fluctuations, and safety [39]. Therefore, in their research, scientists designed an implementation mode using different tools (CAD, 3D tools, PLM, simulation chats, simulation modeling tools, decision making methods, etc.) to create a model that they proposed to apply for a creation shipyard [39]. Researchers in [40], with the aim of connecting the Industry 4.0 technologies to the supply chain, showed that the shipbuilding supply chain should be lean and green, both very important paradigms.

Automotive manufacturers are constantly faced with difficulties in delivering to an on-time schedule due to constant changes in orders and requirements from their customers. Researches proposed a web-based cyber-physical system based on digital twin for abnormal scenarios in automotive industry production lines, and its capability was tested via experiments [41]. Their conclusions were very promising, reaching an average prediction performance of 94% for the production plant, thus confirming that it could be a solution to predicting whether production was possible according to the production plan.

With the exception of using DT for the manufacturing process, there is concern within the automotive industry regarding privacy, because DT in smart cars can collect data—starting from the manufacturing process, through the operational driving stage, the decision-making stage which is based on measurements and historical data, and in the final reporting stage. Considering this, scientists have analyzed the automotive ecosystem from the point of view of privacy problems/anomalies with the intention of reducing risk [42].

Another important area where digital twin can bring considerable improvement is in the energy sector. As the electricity sector is one of the main polluters when it comes to greenhouse gas emissions, it is necessary to assess the situation and see how digital twin and smart manufacturing can bring value [43]. By using digital twin to create a way to monitor the energy system, the industry can achieve a better view of the entire process—from modeling a service to customer behavior—which will allow it to achieve a pattern that will lead to a reduction in energy consumption [44].

Regarding the renewable energy sector, the authors of [45] tested digital modeling with the generative power system, with the aim of solving some problems. They used a hybrid renewable energy system that was useful in updating DT in the energy sector, concluding that the components of the architecture were very important for the design of future power systems. Additionally, in [46] the author presented the application of Industry 4.0 for the integration of distributed energy resource applications using a predictive analysis model.

Another area where digital twin is recording impressive results is in the retail industry, where it is being used to improve product delivery and customer service by better analyzing customer behaviors and feelings, products, and other elements. Using proper tools allows retailers to have an overview of the process, exploring the materials, products, customer feedback, sales (local, regional, national, or international), costs, and—not to be forgotten—production time [47].

Creating new revenues and answering strategic questions are the main values that can be created with digital twin. Questions that once had no answer or could not be answered may find a solution using the applications of digital twin.

The main objective of this study was to perform a bibliometric analysis of the articles published in the Web of Science (WoS) database on digital twin and smart manufacturing, and then to determine the extent to which these keywords were in correlation with Industry 4.0. Therefore, the general search focused on digital twin and smart manufacturing, and the specific search focused on digital twin and smart manufacturing and Industry 4.0. For this purpose, the following questions guided this research:

*Q1. How did the publications on digital twin and smart manufacturing evolve? How did they evolve for digital twin and smart manufacturing and Industry 4.0?*

*Q2. Which authors have published the highest number of publications on the subject, and which authors have the highest number of citations?*

*Q3. Which institutions, publishers and countries focused most of their attention on digital twin, smart manufacturing, and Industry 4.0?*

*Q4. What type of documents are most frequently published?*

*Q5. What citation nodes are the most influential in the network map?*

The results showed that the number of published articles has significantly increased in the last two years (2019 and 2020). The author analysis revealed that Tao F. was the author with the highest influence among the scientific community with regard to digital twin and smart manufacturing, but Soderberg R. was the author that most presented the relation between digital twin, smart manufacturing, and Industry 4.0. As we can see from both analyses conducted, it is only in recent times that the subjects have increased in publication, and are rapidly becoming of interest, which can only mean that technologies implemented in the future will be part of these areas. There are some clusters of collaboration between authors, with other clusters still developing, and soon they will all form a network of data and information useful both for the academic community and for the socio-economic environment.

In order to answer all the research questions and achieve the desired objectives, the article is divided into two sections, one for the general search using digital twin and smart manufacturing, and the other for the specific search which included Industry 4.0. The framework is presented, the method of the study is presented, and the results and discussions on the bibliometric analysis are then presented. Additionally, the limitations of the study and some future lines of research are presented, with the article ending with conclusions on the results found.

## 2. Materials and Methods

The researchers considered the structure of bibliometric studies, where a topic is being studied and evaluated, for this paper’s structure and presentation.

To conduct the study, the literature search took place during September 2021 using the Web of Science database. This database was selected as the source of data collection because for researchers and university professors in Romania, this is the platform with the highest degree of stringency and most prestigious work, and its prestige is international. As Web of Science contains the best scientific productions in various fields and the papers from this database have a high impact factor, which is a very important indicator in Romania then this is one of the reasons of choosing it. It also offers, most recently, many features that can be used in searching, selecting and gathering information.

In order to obtain the final samples to analyze, we first began by choosing the keywords needed to conduct the search in the platform ISI Web of Science. The keywords chosen were digital twin and smart manufacturing. In the search field we used ”digital twin ”AND” smart manufacturing”, thus obtaining 397 documents. Second, since we wanted to focus also on Industry 4.0, we added a more specific search where we used the three keywords connected by ”AND”. This search yielded 173 results.

To refine these results, we used some inclusion and exclusion criteria that led us to the samples used for the bibliometric analysis. These criteria were:

- The inclusion criteria:
  - Use of ”digital twin” AND ”smart manufacturing” for the general bibliometric analysis;
  - Use of ”digital twin” AND ”smart manufacturing” AND ”Industry 4.0” for the specific bibliometric analysis;
  - Production of materials up to 2020;
  - Web of Science collection;
- The exclusion criteria:
  - Material published in 2021, due to the fact that the year was not yet complete;
  - Other scientific databases.

**Figure 1:**
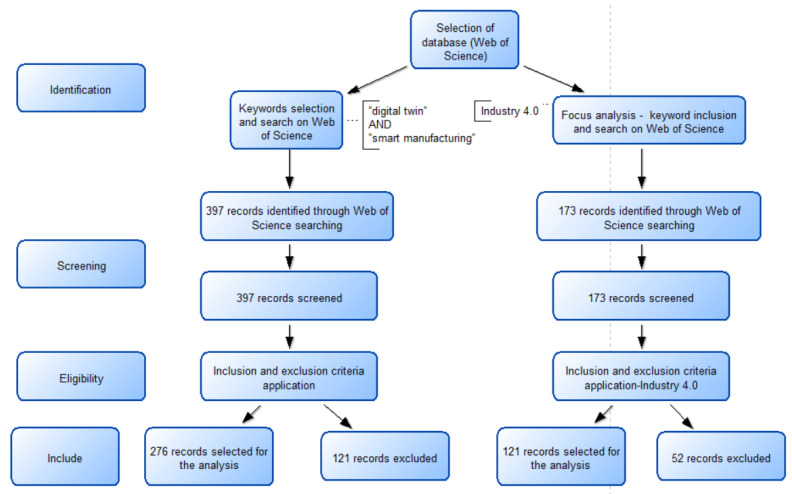
PRISMA flowchart that shows the step-by-step process of the application of inclusion and exclusion criteria that generated the final number of studies for analysis.

After applying these criteria, we obtained 276 materials out of 397 materials for the more general search, and 121 materials out of 173 for the focus of the article. The flowchart regarding the methodology is presented in Figure 1.

The next step was to download all the records in plain text with all the information given: publication type, authors, author keywords, abstract, researcher IDs, times cited, publication date, etc. After this, the downloaded database was verified to see whether it contained duplicate records that needed to be deleted in order to conduct the bibliometric analysis.

To have a bibliometric analysis that is both qualitative and quantitative, we also analyzed the journals in which the materials were published.

The general bibliometric analysis was conducted in the following order: first, the yearly productivity of publications on the subject; second, determination of the most productive authors, with affiliations, citations and impact index in all three areas; third, the most productive institutions; fourth, the publishers with the highest number of publications; fifth, an analysis of the classification of scientific documents; sixth, the countries with the highest number of publications; and then we created the network of citation analysis, using the authors as a unit, with the help of VOSviewer.

## 3. Results

This section presents the results obtained, firstly from the general bibliometric search (digital twin and smart manufacturing), and then the results for the specific search (digital twin and smart manufacturing and Industry 4.0).

### 3.1. General Search: Digital Twin and Smart Manufacturing

As mentioned previously, a total of 276 articles, published in journals, written by 898 authors from 393 institutions and 49 countries, were found. Therefore, the basic bibliometric indicators such as year of publication, most productive authors, journal, institutions and countries, and the connection between descriptors, is presented.

**Figure 2:**
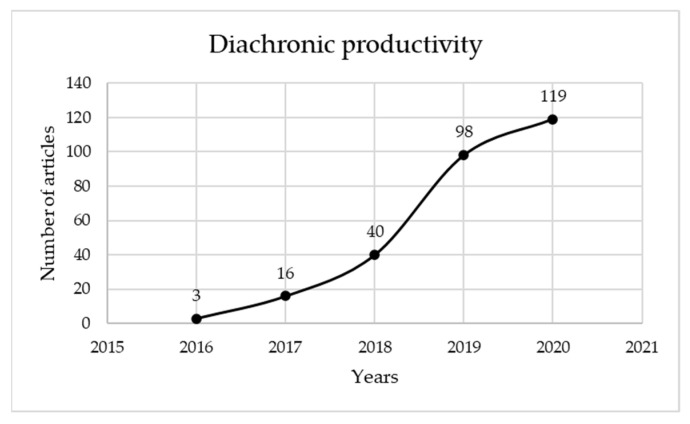
Diachronic productivity of digital twin and smart manufacturing materials published in Web of Science since 2016.

Figure 2 presents how literature has been distributed since the initial presentation of the topic in a material in 2016, until 2020. It can be observed that the last two years, 2019 and 2020, constitute 78.63% of the total scientific production of these five years. This increase demonstrates the interest that digital twin is garnering in industry, and the strong connection to smart manufacturing. The trend seems to indicate a continuous growth in the number of materials published.

A total of 898 authors contributed to publishing at least one article on the subject analyzed. Among them were some researchers that had high productivity in the field such as Tao F. with 12 materials, followed by Soderberg R. with eight materials published, and then five authors with seven materials. These authors and others are presented in Table 1. Additionally, the impact index column in Table 1 shows that Tao F. was not the author with the highest value; that author was Qi Q. L. with 179.5, compared with Tao’s 137.08.

There was a total of 393 institutions, but just five of them stood out in terms of most numerous scientific production, with more than seven publications (65 institutions had six publications, respectively). The institution in the first place was Beihang University with 13 publications, followed by Chalmers University of Technology with 12 publications, and third, Guangdong University of Technology with 10 publications. If we analyze the first 70 universities, we can see that they mostly were from countries such as China and USA. The institutions with the highest number of publications in digital twin and smart manufacturing are presented in Table 2.

The next step, as presented in Table 3, was the journals analysis. A total of 35 journals were found to have published materials from researchers on this subject. The publisher with the highest number of articles published on this topic was Elsevier (89 documents), which also had the highest number of citations. The publisher with the next highest number of articles was IEEE with 59 documents, however, the impact factor of” Proceedings of IEEE” was 10,961 and the ”Article Influence Score” was 4.298, unlike Elsevier where some journals did not have an impact factor and the journals mainly about medicine had the highest impact factor. Table 3 presents the journal publishers that published the most articles, with the number of citations and the impact index.

**Figure 3:**
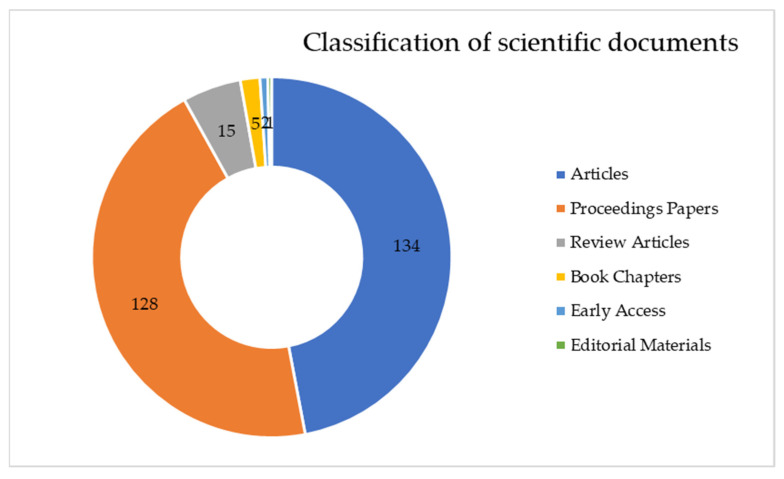
Classification of scientific documents on digital twin and smart manufacturing.

Figure 3 shows the distribution of the different types of scientific publications. The great majority of publications were scientific articles closely followed by proceedings papers.

In order to see which part of the world was most preoccupied with the subject, an analysis of countries in relation to the numbers of publications was undertaken. Table 4 shows the countries with the highest number of publications (≥3 papers). It can be seen that the countries with the highest number of authors who had published at least one material in digital twin and smart manufacturing were China (66), followed by USA (43), and then Italy (31). In countries where one or two articles have been published, we can observe that the subject is becoming a subject of interest, and that development on this topic will increase. 

**Figure 4:**
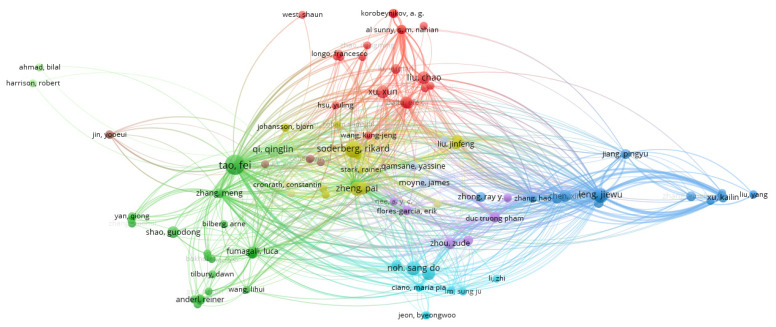
Network of citation analysis using the authors as a unit (general analysis).

Regarding the networks that can be created, we analyzed the network of citation based on the authors (as a unit of analysis). VOSviewer was used to visualize the networks. As can be seen in Figure 3, there were 11 clusters and 1438 links, and the total link strength was 2186. Only authors with a minimum of two documents were analyzed, and of the total number of authors, only 141 met the threshold. Based on all the authors, the largest set of connected items consisted of 120 items (Figure 4).

By conducting an analysis of the resulting clusters, we found that, for example, the largest cluster had 25 items, followed by a cluster of 23 items, and two clusters of 15 items. The main contributors in the first cluster were: Al Sunny, Balta Efe, Barton Kira, Choi Sangsu, Hsu Yuling, Hu Liwen, Korobeynikov, Leu Ming, Liu Chao, Longo Francesco, Lu Yuquian, Ngoc-tu Nguyen, Nicoletti Letizia, Padovano Antonio, Shahriar Rakib, Shukalov, Tilbury Dawn, Wang Kung-Jeng, Wang Wei, West Shaun, Xu Xun, Zakoldaev, Zhao Dongming, Zharinov I., and Zuo Ying. An analysis of the ideas published in the biggest cluster mainly showed a digital twin framework for smart manufacturing, performance monitoring and anomaly detection, service-oriented application for a 4.0 knowledge navigation in the smart factory, virtual factory of the Industry 4.0, and automation, etc., [48,49,50,51,52,53].

The second largest cluster presented digital twin as a comprehensive digital representation of an individual product in which a digitalized product life cycle will play an integral role. Additionally, the authors presented the challenges and constraints that companies face when trying to implement these systems. The communication aspect of digital twin with a number of other applications was shown as a major factor in achieving a complete virtual representation of an asset, process or product. Another element identified as a part of the analysis conducted in this cluster was also the analysis of the symmetry between real and virtual space that enables the analysis of systems under real world conditions. All this could be possible if multiphysics models, sensors and bidirectional data connections between the digital and the physical twin are used [15,17,54,55,56,57,58,59,60].

**Figure 5:**
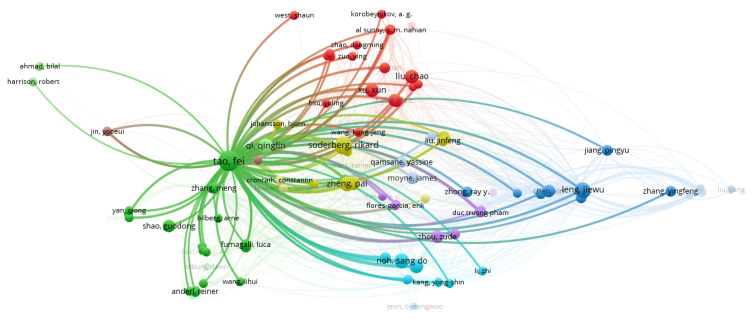
Network of Tao F. citation analysis using the authors as a unit (general analysis).

Figure 5 exemplifies the importance and influence of Tao F. on the subject, which coincides with the previous analysis.

### 3.2. Specific Search: Digital Twin and Smart Manufacturing and Industry 4.0

For the specific analysis, a total of 121 articles were found, published and written by 395 authors, from 205 institutions, and 43 countries. Similar to the general search and analysis, the basic bibliometric indicators such as year of publication, most productive authors, journal, institutions and countries, in addition to the connection between descriptors, is presented.

**Figure 6:**
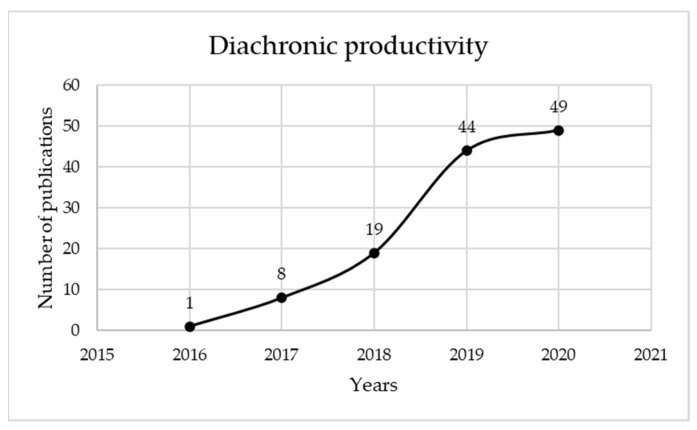
Diachronic productivity of digital twin and smart manufacturing and Industry 4.0 materials published in Web of Science since 2016.

Figure 6 shows how the literature has been distributed since the initial presentation of the topic in a material in 2016, until 2020. It can be observed that, similar to the general search, the latter two years, 2019 and 2020, represented 76.7% of the total scientific production. Although the increase between 2019 and 2020 was not that high as 2018 to 2019, we can still say that there was an increase in publication of the subject among researchers.

A total of 395 authors contributed to publishing at least one article on digital twin and smart manufacturing, and Industry 4.0. Under this criteria, the top researcher, namely, the one with the highest productivity, was Soderberg R. with seven materials. In the general search, he was the second highest author to have published an important number of publications. Lindkvist L., Liu C. and Xu X. were authors with more than six publications who were not present in the first search. Regarding citations, Tao F. was also in first place with an impact index of 221, compared with the next author, of just 86.75, as it can be seen in Table 5. 

Table 6 shows the affiliations of the authors. Only a few of the 205 institutions stood out with more than four publications on the subject. The institution with the most numerous scientific production was Chalmers University of Technology with eight publications, followed by Beihang University and Polytechnic University of Milan, each with five publications. The institutions with the highest number of publications in digital twin and smart manufacturing, and Industry 4.0, are presented in Table 2. There were 174 institutions with just one material.

Similar to the general search and analysis, the next step, as presented in Table 7, refers to the publishers. Again, Elsevier was the publisher with the highest number of publications (40), followed by IEEE with 27 publications. The difference between this and the earlier analysis is that MDPI is now in fourth place, and Taylor & Francis is in fifth, while previously for the general analysis it was the other way around. Although Springer Nature was in third place, MDPI had a higher impact index and, respectively, H-index. Table 7 presents the publishers that published the most articles, with the number of citations and the impact index.

**Figure 7:**
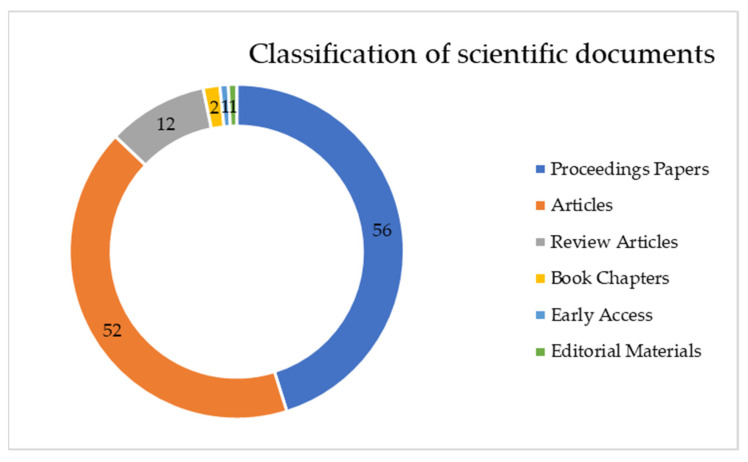
Classification of scientific documents on digital twin and smart manufacturing, and Industry 4.0.

Scientific documents were also classified for the specific analysis (Figure 6). Proceedings papers and articles represented 89.25% of the scientific papers published by researchers. Figure 7 presents the classification based on the number of publications.

Table 8 presents the countries with the highest number of publications (≥4 papers) in relation to the number of publications. The countries with the highest number of authors that published at least one material in digital twin and smart manufacturing and Industry 4.0 were from Italy (18) and China (18), followed by an identical number of documents from authors in Germany, Sweden and USA (12, respectively). 

**Figure 8:**
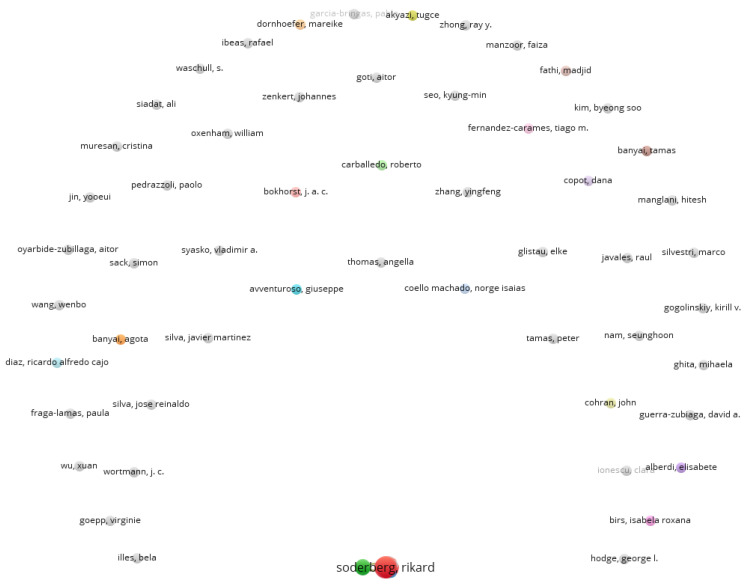
Network of citation analysis using the authors as a unit (specific analysis).

**Figure 9:**
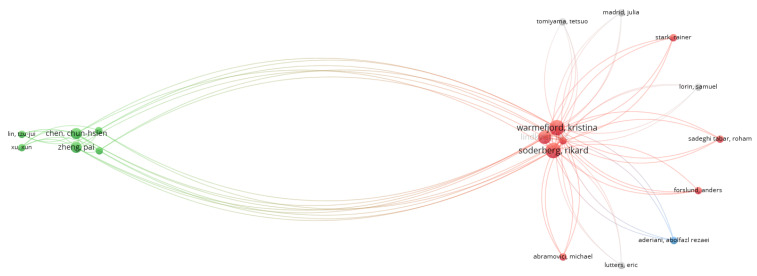
Network of citation analysis using the authors as a unit, showing the main cluster (specific analysis).

The network of citations based on the authors (as a unit of analysis) was also analyzed for the specific analysis. VOSviewer was used to visualize the networks. As shown in Figure 8, there were 60 clusters and 71 links, and the total link strength was 90. Again, only authors with a minimum of two documents were analyzed, and from the total number of authors only 72 met this threshold. From this analysis, the largest set of connected items is presented in Figure 9.

As observed from this cluster, the most influential connection was between the authors Kristina Warmefijord and Rikard Soderberg. Additionally, their knowledge extends to Zheng Pai and Chen Chun-Hsien. The research in the field also presents a collaboration between Kristina Warmefijord and Rikard Soderberg, in which they study digital twin-driven production mainly regarding assembly lines [61], try to find an effective method for spot welding sequence optimization [62], or try to identify industrial challenges in areas such as management, system-level, education, working process and simulation [63]. The findings in the cluster are in accordance with the analysis in Table 5 where Sodenberg R., Warmefjord K. and Lindkvist L. were the authors that had the highest number of publications in digital twin, smart manufacturing and Industry 4.0.

## 4. Discussion

This research facilitates the evolution of scientific production in digital twin, smart manufacturing, and Industry 4.0. These subjects were the most discussed topic of 2021, therefore, the selection was based on technological evolution and where we are heading to. Although the first publication in this field was in 2016, the evolution of publication is on an ascending trend. In general, research shows the strong connection between digital twin, smart manufacturing, and Industry 4.0. The growth in literature on this subject is essentially due to technological development in all areas [64,65]. Conducting this analysis, we can observe how studies were carried out during this time in terms of evolution. Therefore, the research questions stated in the beginning can now be discussed.

*Q1. How did the publications on digital twin and smart manufacturing evolve? How did they evolve for digital twin and smart manufacturing and Industry 4.0?*

Regarding the evolution of the publications, the subject only started to emerge in scientific literature five years ago. Since its first appearance, many researchers have presented and shown the way the subject has evolved in various fields and different industries. A rapid growth was seen in 2019 and 2020, with these years accounting for more than 90% of the materials published over the five years [66,67]. The same pattern can be seen for the specific search, where 2019 and 2020 also hold the record for the years with the highest number of publications. Additionally, this research question highlights the evolution of the subject in the digital world by demonstrating that interest is continually growing among researchers and scientists worldwide. An increase in the number of publications, as demonstrated by the number of publications that were eliminated for the year 2021, show that digital twin, smart manufacturing and Industry 4.0 are the motors of change, because they provide a link between the physical and digital world that is comprehensive and real. Applications of the lifecycle of industrial products can be performed through research, due to engineering software and digitalized equipment [30]. Additionally, it could be observed that the evolution of the subject brought with it research papers that focused on the connection between real and virtual systems, and the opportunities, benefits, and challenges that this technology brings. This is consistent with the findings of [68,69].

*Q2. Which authors have published the highest number of publications on the subject, and which authors have the highest number of citations?*

In relation to the most important authors within the field of study proposed, Tao F. was the author with the highest number of publications (12) and citations (1645) in the field of digital twin and smart manufacturing, while the author with the highest number of publications (7) and just 194 citations in the field of digital twin and smart manufacturing and Industry 4.0 was Soderberg R. He was also the author with the second highest number of publications for the general search. Although Tao F. was not the highest author in the specific search, he still had the highest number of citations (884). However, based on the three keywords used, the most influential author was Tao F. from Beihang University. This analysis was strongly connected with China, which had the greatest number of publications on this topic. Considering the evolution in the field, the author analysis represents the interest of scientists in the field, that the identified authors will be highly cited and also will continue to lead the scientific community in the area of digital twin, smart manufacturing and Industry 4.0. Therefore, whether we are talking about digital twin from the point of view of real vs. virtual systems, manufacturing processes or automation in manufacturing, it can be seen that Tao F. was one of the main contributors. This is also in agreement with [68,69] which mentioned some important contributions of Tao F. such as Digital twin-driven product design, manufacturing and service with big data, Digital Twin Shop-Floor: A New Shop-Floor Paradigm Towards Smart Manufacturing, Digital Twin and Big Data Towards Smart Manufacturing and Industry 4.0: 360 Degree Comparison, etc., [15,17,70].

*Q3. Which institutions, publishers and countries focused most of their attention on digital twin, smart manufacturing, and Industry 4.0?*

Chalmers University of Technology and Beihang University stood out in both the general and specific searches as institutions having the most productive authors in this area. Thus, we can say that they were the most influential institutions on the subject. The cumulated percentage covered by these institutions was over 9% for both searches. Analysis of the top institutions showed that the research conducted by scientists was performed in institutions that placed a great importance in providing research infrastructure. Thus, this analysis shows that investment in technology within institutions will represent an important part in the evolution of digital twin, smart manufacturing and Industry 4.0.

Analysis of the publishers that stood out in terms of publishing the largest number of articles showed Elsevier in first place, and IEEE in second place, with a cumulated percentage of over 79% of the total number of publications. For both searches and analyses, Elsevier, IEEE and Spring Nature were the top three journals that had the highest number of documents published. A change was observed for the fourth and fifth place, that reversed between the general and specific searches (in the general search, Taylor & Francis were fourth and MDPI fifth, while in the specific search, MDPI was fourth and Taylor & Francis fifth). Considering that only ISI Web of Knowledge was analyzed, it is possible that other publishers may have achieved first place if we had introduced a larger number of scientific platforms. The analysis of publishers was selected as being important, as authors tend to choose publishers with high recognition among the scientific community.

The countries with the greatest number of publications were China (first place), USA (second place) and Italy (third place) in the general search, while for the specific search, China and Italy equally occupied first place, followed by Germany and USA equally being in second place. If correlated with the citations analysis, China remained in first place. The most productive institution was from China, which is in accordance with [71].

*Q4. What type of documents are most frequently published?*

The two most frequently used types of material observed from the analysis were ”articles” and ”proceeding papers”, comprising over 78% of materials for both searches, and therefore, a substantial majority of the total number of publications. Since these types cover most of the field, other types of document categories do not have sufficient representation to warrant mentioning their important role for the subject in the scientific literature. This parameter was important in the analysis because for long time the “proceeding paper” had a different understanding, and was assigned within the WoS database to journal articles that initially were conference papers. They were later assigned two labels, articles and proceedings papers, since analysis revealed differences between the number of pages and citations, thus giving us the chance to also differentiate them in this paper. It was seen that these types are the preferred manner in which scientists/researchers choose to present their findings to the scientific world [72].

*Q5. What citation nodes are the most influential in the network map?*

After conducting the analysis using VOSviewer, it was observed that the network revolved around Tao F. for the general search, with other authors just starting to collaborate with the created clusters. This demonstrated that Tao F. is currently the author with the highest influence on this subject. Additionally, the specific search revealed that Sodenberg R. is the author with the strongest connection of the three keywords used in this paper. The analysis of citation nodes was performed because the frequency of citations constitutes an academic value and impact mark. Thus, in our networks, we could see the most influential authors and their importance in the field based on the number of citations and links created.

Further analysis of the documents from the selected articles showed that there were some elements that are closely connected to the concept of digital twin, such as physical entity, virtual entity, real and virtual environment, connection between real to virtual, twinning, advantages, disadvantages, challenges, DT and product life-cycle, data, implementation, and virtual entities integration, etc., [15,26,70,73,74,75,76,77,78,79,80,81,82,83]. All mentioned references, and many others, describe these elements as themes, with some points being addressed and presented. For example, one important element discussed the parameters from the real environment that need to be addressed in the virtual environment in order to have a solid connection between them [84,85,86,87]. These parameters are also in accordance with [69] because they found similar themes that they approached, along with future research directions.

With the exception of answering the questions, increasing productivity and employee efficiency can be achieved using devices utilized in the smart industry. Digital twin has the ability to provide an edge-to-cloud architecture that will contribute to the optimization of operational costs. Until 2019, digital twin concepts and smart manufacturing were mainly applied in industries from aerospace to naval [71].

The progress in technology, along with the COVID-19 pandemic, caused companies to rethink their future plans due to unpredictable times, leading to businesses starting to build intelligent twins, creating mirrored environment [72]

Scientists have also analyzed the terminology and framework of digital twin, smart manufacturing and Industry 4.0, showing, therefore, that the standards are only now just being established. For example, the International Organization for Standardization (ISO) is currently developing:

- The concept and terminology (IEC AWI 30173);
- The manufacturing framework (ISO/FDIS 23247-1);
- The framework for health and well-being in smart cities (IEEE 11073) [88,89].

Future research is needed in novel sensors, which has not been sufficiently explored and will bring benefits related to 5G communication [90].

Another possible area for research within the field of digital twin and smart manufacturing, where the vision of application is not just for the next period but for the next decade or more, is healthcare. Although some technology exists (digital twin in cardiology), collaboration between the healthcare industry and scientific knowledge will contribute to the exploration of other healthcare problems, leading to personalized medicine in the future [91,92,93].

Also, digital twin technology is still at its beginning in agriculture and construction [94]. Regarding agriculture, digital twin technology can assist/improve storing and collecting data, categorize different activities related to workflow, analyze data, apply/learn/measure the content and capacity of the soil, conduct a simulation regarding crops and their outcomes, prediction weather for future productivity, and analyze invasive factors, etc., [95].

Considering the findings, synchronization is the main element that contributes to the development of DT technologies. The aspect that is intensively studied in many areas is that of being able to synchronize the reality with the virtual space, to gather all the data and properly use them [96]. Linking not just real to virtual, but in addition, linking elements/entities inside the virtual space is also a challenge due to the fact that proper interactions need to be established for the digital twin platform to work.

There are studies that focused on the technical challenges that come along with digital twin technology development, but unfortunately there are still areas where research is needed, such as the user’s point of view when discussing the attitude, experience or even acceptance of digital twin technology. There are elements, such as users’ personality, that need to be considered when using digital twin technology, because this can have an influence on the product design, production planning, and the decision-making process, etc., [97,98,99,100].

A challenge for the development of digital twin is the creation of a platform that integrates and connects different systems that already exist. A framework that incorporates all dates without resulting in inconsistencies would help build an integrated digital twin platform necessary for the economic environment, regardless of its size (SMEs or large companies) [101,102].

Smart manufacturing is not possible without proper integration of the elements mentioned, and a common understanding (about the domain, process, production, application, and requirements) is the basis for future development [103].

Manufacturing industries such as aerospace, automotive and power generation are still investing in research to construct a digital twin that has all the capabilities required in physical space, in the virtual space, using digital twin technologies [18,104].

A very important aspect that needs to be considered regarding the challenges is that uncertainty and the ever-changing environment are factors that are common to manufacturing industries due to the differences in the customer demands, technology evolution and product design. Thus, digital twin technology used in smart manufacturing needs to keep in mind that we are dealing with a very complex system, so the ability to be flexible is highly important [105].

Additionally, digital twin should be able to evolve in linking engineering, economic, technological data and any other challenges that the manufacturing industries face. The real challenge is to allow enough time and money for the development and implementation of digital twin technology for smart manufacturing [106].

Study Limitations

For a high quality bibliometric analysis, it is necessary to mention its limitations.

First, the analysis was performed only using WoS, therefore further studies should also address other databases.

Second, this study had a quantitative point of view, presenting the productivity and the impact of the material published. Future bibliometric analysis can be performed with a focus on the qualitative point of view, by selecting important identified themes to explore and discuss, selecting an industry, and analyzing from both points of view the evolution of an industry in correlation to a theme and the innovation on the subject, etc.

Therefore, we present a strong connection in technology between digital twin, smart manufacturing, and Industry 4.0. Additionally, the bibliometric maps/networks can be extended beyond the current study, to show networks of co-authors, co-words, etc.

## 5. Conclusions

The bibliometric analysis used in this paper represents a tool for researchers to observe the current state on a selected topic.

The aim of this study was to show the ever-changing technology and its rapid evolution by exemplifying the topics most currently discussed in industry: digital twin, smart manufacturing and Industry 4.0. A considerable evolution of this subject was observed to have occurred since 2019, which demonstrates that research and innovation on the subject have just started to increase. 

Digital twin is an important part of future technology development that will influence the world’s industries as we know them today. Thus, it is necessary to understand the connection between digital twin, smart manufacturing and Industry 4.0, to be able to further innovate and improve the working process in all industries.

This article was inspired by the existing facts of the subject, in order to present the ongoing evolution of this topic based on scientific literature.

Considering that all three fields of study are currently in full development, more studies are needed to contribute to scientific literature on this topic. Additionally, topics such as agriculture, healthcare, 5G communication and construction need more research in order to apply a technology that can answer realistic problems and can move these domains to the next level of evolvement. To have a worldwide view on the subject, researchers from different countries should engage in highly productive collaborations.

## Figures and Tables

**Table 1:** Most productive authors (≥6 papers), with affiliations, citations and impact index in digital twin and smart manufacturing.

Author	No. Doc	%	Citations in WoS Core	Impact Index	Affiliations
Tao F.	12	0.4348	1645	137.08	Beihang University
Soderberg R.	8	0.0289	196	24.5	Chalmers University of Technology
Leng J. W.	7	0.0253	423	60.43	Guangdong University of Technology
Liu Q.	7	0.0253	423	60.43	Guangdong University of Technology
Parlikad A. K.	7	0.0253	54	7.71	University Cambridge
Warmefjord K.	7	0.0253	194	27.71	Chalmers University of Technology
Xie X.	7	0.0253	42	6	University Cambridge
Lu Q. C.	6	0.0217	54	9	UCL
Noh S. D.	6	0.0217	66	11	Sungkyunkwan University
Qi Q. L.	6	0.0217	1077	179.5	Beihang University
Zheng P.	6	0.0217	212	35.33	Nanyang Technology University
6 researchers	5	-	-	-	-
10 researchers	4	-	-	-	-
22 researchers	3	-	-	-	-
104 researchers	2	-	-	-	-
745 researchers	1	-	-	-	-

**Table 2:** Most productive institutions (≥7 papers), with citations and impact index in digital twin and smart manufacturing.

Affiliations	No. Doc	%	Citations	Impact Index
Beihang University	13	0.047	1648	126.77
Chalmers University of Technology	12	0.043	267	22.25
Guangdong University of Technology	10	0.036	455	45.5
Sungkyunkwan University SKKU	7	0.025	106	15.14
University of Cambridge	7	0.025	104	14.85
7 institutions	6	-	-	-
6 institutions	5	-	-	-
4 institutions	4	-	-	-
21 institutions	3	-	-	-
59 institutions	2	-	-	-
291 institutions	1	-	-	-

**Table 3:** Publishers with the highest number of publications (≥3 papers) with citations and impact index in digital twin and smart manufacturing.

Publisher	No. Doc	%	Citations	Impact Index	H-Index
Elsevier	89	32.24	2451	27.54	25
IEEE	59	21.37	1348	22.85	13
Springer Nature	46	16.66	1273	27.67	14
Taylor & Francis	19	6.88	390	20.53	9
MDPI	14	5.07	270	19.29	8
American Society of Mechanical Engineers	7	2.54	12	1.71	2
Iop Publishing Ltd.	4	1.45	12	3	1
American Society for Testing and Materials	3	1.09	6	2	2
Korean Society for Precision Engineering	3	1.09	13	4.33	2
Sage	3	1.09	14	4.67	2
4 journals	2	-	-	-	
21 journals	1	-	-	-	

**Table 4:** Countries with the highest number of publications (≥3 papers) with citations and impact index in digital twin and smart manufacturing.

Country	No. Doc	%	Citations	Impact Index	H-Index
China	66	23.913	3147	47.68	26
USA	43	15.580	541	12.58	11
Italy	31	11.232	534	17.23	12
Germany	24	8.696	388	16.17	9
South Korea	22	7.971	158	7.18	7
Sweden	21	7.609	382	18.19	8
England	20	7.246	306	15.3	9
Singapore	15	5.435	598	39.87	8
Russia	8	2.899	20	2.5	2
Austria	7	2.536	39	5.57	2
5 countries	6	-	-	-	
3 countries	5	-	-	-	
5 countries	4	-	-	-	
5 countries	3	-	-	-	
6 countries	2	-	-	-	
15 countries	1	-	-	-	

**Table 5:** Most productive authors (≥4 papers), with affiliations, citations and impact index in digital twin and smart manufacturing and Industry 4.0.

Author	No. Doc	%	Citations in WoS Core	Impact Index	Affiliations
Soderberg R.	7	5.785	194	27.71	Chalmers University of Technology
Warmefjord K.	7	5.785	194	27.71	Chalmers University of Technology
Lindkvist L.	4	3.306	166	41.50	Chalmers University of Technology
Liu C.	4	3.306	231	57.75	Cardiff University
Tao F.	4	3.306	884	221.00	Beihang University
Xu X.	4	3.306	347	86.75	University Auckland
5 researchers	3				
30 researchers	2				
354 researchers	1				
745 researchers	1	-	-	-	-

**Table 6:** Most productive institutions (≥3 papers), with citations and impact index in digital twin and smart manufacturing, and Industry 4.0.

Affiliations	No. Doc	%	Citations	Impact Index
Chalmers University of Technology	8	6.612	198	24.75
Beihang University	5	4.132	889	177.8
Polytechnic University of Milan	5	4.132	310	62
Guangdong University of Technology	4	3.306	176	44
University of Auckland	4	3.306	347	86.75
Aalborg University	3	2.479	51	17
Nanyang Technological University	3	2.479	190	63.33
National Institute of Education Singapore	3	2.479	190	63.33
RWTH Aachen University	3	2.479	3	1
22 institutions	2			
174 institutions	1	-	-	-

**Table 7:** Publishers with the highest number of publications (≥2 papers) with citations and impact index in digital twin and smart manufacturing and Industry 4.0.

Publisher	No. Doc	%	Citations	Impact Index	H-Index
Elsevier	40	33.058	1636	40.9	18
IEEE	27	22.314	1069	39.59	9
Springer Nature	17	14.050	198	11.65	5
MDPI	10	8.264	151	15.1	7
Taylor & Francis	5	4.132	47	9.4	3
American Society of Mechanical Engineers	4	3.306	0	0	0
Asme	2	1.653	3	1.5	1
Iop Publishing Ltd.	2	1.653	0	0	0
Sage	2	1.653	12	6	2
12 journals	1	-	-	-	

**Table 8:** Countries with the highest number of publications (≥4 papers) with citations and impact index in digital twin and smart manufacturing, and Industry 4.0.

Country	No. Doc	%	Citations	Impact Index	H-Index
China	18	14.876	1302	72.33	13
Italy	18	14.876	430	23.89	9
Germany	12	9.917	259	21.58	6
Sweden	12	9.917	338	28.17	6
USA	12	9.917	67	5.58	5
Singapore	9	7.438	532	59.11	5
Hungary	5	4.132	22	4.4	3
New Zealand	5	4.132	347	69.4	4
Russia	5	4.132	14	2.8	1
South Korea	5	4.132	36	7.2	3
Brazil	4	3.306	57	14.25	3
Denmark	4	3.306	60	15	3
6 countries	3	-	-	-	
10 countries	2	-	-	-	
15 countries	1	-	-	-	

## Data Availability

Not applicable.
